# Hospital-Based Palliative Care: Quality Metrics That Matter

**Published:** 2015-11-01

**Authors:** Rhonda Gradwohl, Jeannine M. Brant

**Affiliations:** Billings Clinic, Billings, Montana

Recent data indicate that practitioners incorporate the latest medical evidence into their treatment decisions only 50% of the time, preferring to practice what they are comfortable with (Trusko, Pexton, Harrington, & Gupta, 2007). The result has been an increased spotlight on standardizing care and measuring outcomes to improve health-care quality.

Quality-improvement metrics are essential for driving hospital quality and measuring the effectiveness of quality programs. Hospital quality and outcomes can be measured using a variety of indicators that provide unique perspectives on quality of care. In the past, determinants of quality generally included clinical outcomes data and patient-reported experiences in the hospital.

More recently, hospital quality is reflective of adherence to evidence-based practice and is often defined and monitored by regulatory bodies such as the Joint Commission (JC) and the Centers for Medicare and Medicaid Services (CMS), which provide a more refined, less varied definition of quality. Indicators are also often determined by professional organizations, as standards are set by the experts themselves within the given organization. Overall, hospitals have a responsibility to evaluate ongoing data to ensure that safe, evidence-based, and quality care is consistently delivered. Quality care in the hospital has been linked to survival, functional ability, successful care transitions, and quality of life (CMS, 2015).

A plethora of metrics exists to evaluate hospital-based quality care, but not all are related to quality cancer and palliative care. For example, the majority of CMS measures focus on cardiac care, pneumonia, postsurgical care, and mortality. However, in some ways, the CMS initiatives have driven quality measures in other disease states and for other hospital-based care including palliative care. This article will highlight hospital-based palliative care metrics measured in the article by Humphreys and Harman (2014) and discuss other metrics amenable for measuring palliative care programs (Humphreys & Harman, 2014).

## PALLIATIVE CARE IN THE HOSPITAL

Palliative care is increasingly becoming an integral component of quality hospital care. The American Society of Clinical Oncology suggests that palliative care should be integrated into oncology care (Smith et al., 2012). Unfortunately, palliative care has been used synonymously with end-of-life care, but clinicians need to understand the more global definition—the relief of suffering for all patients.

In the hospital setting, patients with cancer are commonly admitted for uncontrolled symptoms related to cancer treatment, the disease itself, and other comorbid conditions (Yennurajalingam et al., 2012). Studies reveal that early palliative care can improve quality of life and even prolong survival (Temel et al., 2010).

As hospitals develop palliative care programs, attention should be given to setting up metrics to measure the success of the palliative program and to use data to drive ongoing quality appraisal and improvement. It is important to keep in mind that the metrics may be geared toward four separate audiences: payers, providers/nurses, patients/families, and administrators, whose views on quality care are diverse (Table 1). Gathering metrics for each group is essential in measuring a program that satisfies all vested audiences.

**Table 1:**
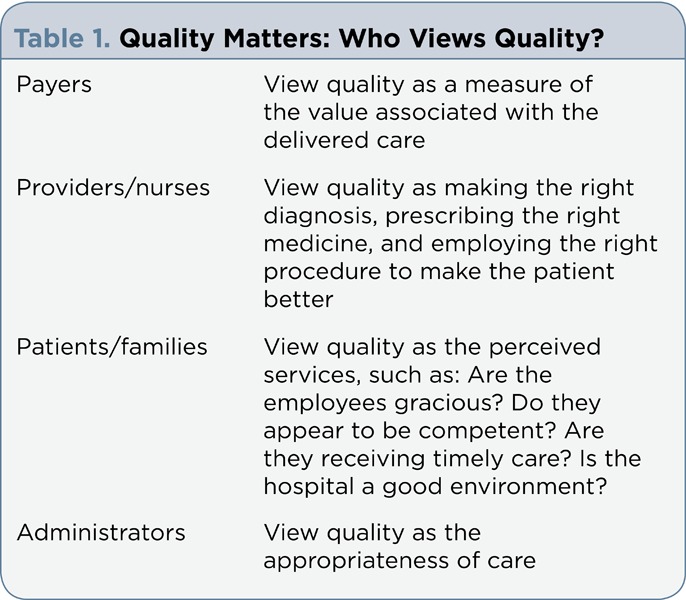
Quality Matters: Who Views Quality?

## OVERVIEW OF STUDY BY HUMPHREYS AND HARMAN

The primary goal of the Humphreys and Harman study discussed by Regina Fink beginning on page 597 was to examine health outcomes for patients who were referred to early vs. late palliative care and then to better define late-referral patients in terms of length of hospital stay and in-hospital mortality (Humphreys & Harman, 2014). Length of hospital stay (LOS) and hospital mortality data are automatically collected for hospitalized patients through CMS requirements and can be valuable outcome measures of a palliative care program’s success. One would anticipate seeing a lower length of stay with those on palliative care than those not on a palliative care service, as symptoms could potentially be managed more quickly by a team of experts, and mortality should potentially decrease as patients are transitioned out of the hospital for supportive care rather than lifesaving measures provided in the acute setting.

A 𝝌2 or Student’s t-test was used to compare LOS and mortality between patients referred early or late to the palliative care service. The challenge with these analyses is parsing out groups appropriately within the organization and defining early vs. late groups. In this study, those referred within a week of admission were considered early, and those referred after a week were considered late. Both outcomes were in support of earlier palliative care leading to better outcomes (Humphreys & Harman, 2014).

## METRICS TO MEASURE PALLIATIVE CARE

The Center to Advance Palliative Care (CAPC) has led to the development of specific metrics to measure the quality of palliative care. These metrics provide a standardized methodology to collect and analyze data prospectively for both quality improvement and research purposes.

Consensus recommendations for consultation services were disseminated in 2008; metrics for inpatient services, in 2009; and metrics for clinical care and customer satisfaction, in 2010 (Weissman & Meier, 2008, 2009; Weissman, Morrison, & Meier, 2010). These metrics are commonly referred to as the gold-standard measurements for palliative care. Similar to CMS measures from Table 1, metrics are categorized into operational metrics (e.g., consultation date, diagnosis, LOS), clinical metrics (e.g., symptoms), customer metrics (e.g., patient satisfaction), and financial metrics (e.g., hospital costs, case-mix index).

As mentioned, defining and standardizing hospital-based metrics and describing how each is measured are critical for standardization, so benchmarks can be established and studies can be compared. In the Humphreys and Harman study, LOS and mortality are both clearly defined according to CMS guidelines, which also define readmission measures, complication measures, and some measures of patient satisfaction (CMS, 2015). Both CMS measures and CAPC measures should be referred to for overall analysis of data.

Table 2 provides a list of the most commonly used palliative care outcome metrics, a definition of each metric, and a method of calculation. In addition to these outcome measures, process measures can also be used to evaluate program components such as documentation of symptoms, care and goals of treatment, support provided to patients and caregivers, and the transition plan.

**Table 2:**
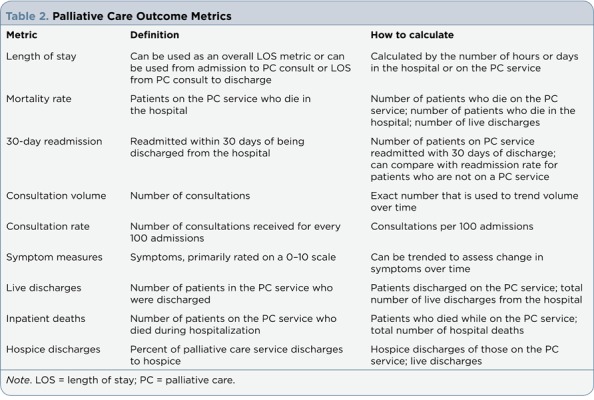
Palliative Care Outcome Metric

More recently, a team of experts from the American Academy of Hospice and Palliative Medicine (AAHPM) and the Hospice and Palliative Nurses Association (HPNA) embarked on a consensus project called Measuring What Matters (MWM), which was published in the *Journal of Pain and Symptom Management* (Dy et al., 2015). The 10 measures, identified from an initial set of 75 measures, are recommended for measurement to benchmark best practices. The measures are balanced to examine physical, psychological, social, spiritual, and program metrics, which comprise all domains of care (Table 3).

**Table 3:**
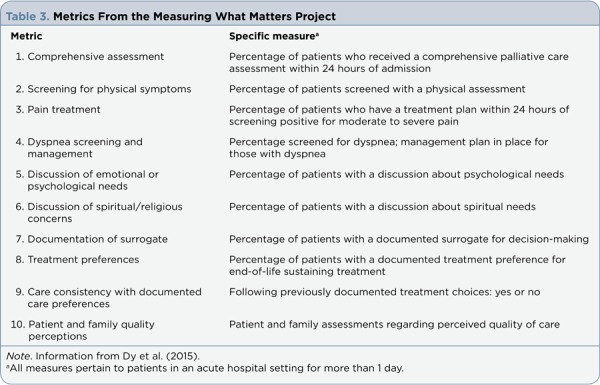
Metrics From the Measuring What Matters Project

**Patient-Satisfaction Metrics**

Patient and family satisfaction is an important component of any program. Unfortunately, patients are generally uninformed about the quality of service and the acceptable standard of care they will receive in the hospital setting (Trusko et al., 2007). However, patients are now being asked more frequently to be involved in their care and to provide feedback about their care experience.

CMS uses the Hospital Consumer Assessment of Healthcare Providers and Systems (HCAHPS) to garner patient satisfaction, and some questions may indirectly relate to palliative care satisfaction (HCAHPS, 2015). For example, two pain measures exist: (1) During this stay, how well was your pain well controlled? (2) During this stay, how often did the staff do everything it could to help you with your pain? Questions about care transitions may also reflect involvement of the palliative care service, which could help provide a seamless delivery of care.

The HCAHPS survey is directly tied to reimbursement through CMS; therefore, high motivation exists for hospitals to achieve high satisfaction scores. But more specific patient and family satisfaction tools exist for palliative care such as the FAMCARE Scale (Can et al., 2011; Teresi, Ornstein, Ocepek-Welikson, Ramirez, & Siu, 2014). Hospitals should be encouraged to use these tools to explore palliative care services in a more holistic and in-depth manner.

## CONCLUSION

Definitions of quality in the hospital setting are often complicated and fluid. In fact, confusion regarding a clear definition of quality is often what impedes timely quality initiatives (Trusko et al., 2007). Therefore, using a set of gold-standard measures such as those set by CAPC can help palliative care programs stay focused on preidentified outcomes. "Quality medicine is doing the right things to the right people in the right place at the right time in an efficient and cost-effective manner" (Trusko et al., 2007, p 27).
